# Supplementary material for: Treatment effect heterogeneity following type 2 diabetes treatment with GLP1-receptor agonists and SGLT2-inhibitors: a systematic review
Source: Commun Med (Lond). 2023 Oct 5;3:131. doi: 10.1038/s43856-023-00359-w (PMC10551026; doi:10.1038/s43856-023-00359-w)

Supplementary Information for:

Treatment effect heterogeneity following type 2 diabetes treatment with GLP1-receptor agonists and SGLT2-inhibitors: a systematic review

Authors: Katherine G Young, Eram Haider McInnes, Robert J Massey, Anna R Kahkoska, Scott J Pilla, Sridharan Raghavan, Maggie A Stanislawski, Deirdre K Tobias, Andrew P McGovern, Adem Y Dawed, Angus G Jones, Ewan R Pearson, John M Dennis, ADA/EASD PDMI

Supplementary Note 1

Search terms

GLP1RA

#1

"Diabetes Mellitus, Type 2"[Mesh] OR TD2[Title/Abstract] OR "Type 2 diabetes"[Title/Abstract] OR "Diabetes, type 2"[Title/Abstract

#2

GLP1RA OR "GLP1 Receptor Agonists" OR "GLP-1 Receptor Agonists" OR "GLP1RA" OR Exenatide OR Liraglutide OR Lixisenatide OR Semaglutide OR Dulaglutide OR Albiglutide

#3

White[Title/Abstract] OR Caucasian[Title/Abstract] OR Asian[Title/Abstract] OR African[Title/Abstract] OR Ethnicity[Title/Abstract] OR Ethnic[Title/Abstract]

#4

Age[Title/Abstract] OR "Diabetes duration"[Title/Abstract] OR "BMI"[Title/Abstract] OR "Body Mass Index"[Title/Abstract] OR Adiposity[Title/Abstract] OR "Sex"[Title/Abstract] OR "clinical features"[Title/Abstract]

#5

"Insulin resistant"[Title/Abstract] OR "Insulin deficient"[Title/Abstract] OR "beta-cell function"[Title/Abstract] OR "c-peptide*"[Title/Abstract] OR "metabolite*"[Title/Abstract] OR "Metabolomic"[Title/Abstract] OR "Proteomic"[Title/Abstract] OR "Protein*"[Title/Abstract] OR "peptide*"[Title/Abstract]

#6

(Genotype[Title/Abstract] OR SNP[Title/Abstract] OR variant[Title/Abstract] OR GWAS[Title/Abstract] OR "Genome Wide Association Study"[Title/Abstract] OR Genetic[Title/Abstract] OR Epigenetic[Title/Abstract] OR Methylation[Title/Abstract]) OR ((("Genotype"[Mesh]) OR "Genome-Wide Association Study"[Mesh]) OR "Methylation"[Mesh])

#7

Precision[Title/Abstract] OR Prediction[Title/Abstract] OR Stratification[Title/Abstract] OR Stratified [Title/Abstract] OR Individualised[Title/Abstract] OR Individualisation[Title/Abstract] OR Personalized[Title/Abstract] OR Personalization[Title/Abstract] OR targeted[Title/Abstract]

#8

RCT OR randomis* OR randomiz*

#9

#3 OR #4 OR #5 OR #6 OR #7

OUTCOME: HBA1C

#10

"hba1c reduction"[Title/Abstract] OR "hba1c change*"[Title/Abstract] OR "hba1c response"[Title/Abstract] OR "treatment response"[Title/Abstract] OR "glycaemic efficacy"[Title/Abstract] OR "glycemic efficacy"[Title/Abstract] OR fall in hba1c[Title/Abstract] OR change from baseline in hba1c[Title/Abstract] OR change in hba1c[Title/Abstract] OR changes in hba1c[Title/Abstract] OR slope of change[Title/Abstract] OR "hba1c outcome*"[Title/Abstract] OR outcome hba1c[Title/Abstract] OR "A1C change*"[Title/Abstract] OR "A1C reduction"[Title/Abstract] OR A1C response[Title/Abstract] OR A1C outcome*[Title/Abstract]

#11

#1 AND #2 AND #9 AND #10

OUTCOME: CARDIOVASCULAR DISEASE

#12

((((((((("Cardiovascular Diseases"[Mesh]) OR "Myocardial Infarction"[Mesh]) OR "Myocardial Revascularization"[Mesh]) OR "Acute Coronary Syndrome"[Mesh]) OR "Myocardial Ischemia"[Mesh]) OR "Heart Failure"[Mesh]) OR "Angioplasty"[Mesh]) OR "Percutaneous Coronary Intervention"[Mesh]) OR "Stroke"[Mesh]) OR "Angina Pectoris"[Mesh])

#13

"cardiovascular disease"[Title/Abstract] OR "myocardial infarction"[Title/Abstract] OR revasculari*[Title/Abstract] OR "acute coronary syndrome"[Title/Abstract] OR "coronary heart disease"[Title/Abstract] OR "heart failure"[Title/Abstract] OR "cardiac failure"[Title/Abstract] OR angioplasty OR "percutaneous coronary intervention"[Title/Abstract] OR "cardiovascular mortality"[Title/Abstract] OR mace OR stroke OR angina OR "major adverse cardiovascular event*"[Title/Abstract] OR chd[Title/Abstract] OR acs[Title/Abstract] OR mi[Title/Abstract] OR hf[Title/Abstract] OR pci[Title/Abstract] OR cvd[Title/Abstract] OR sbp[Title/Abstract] OR dpb[Title/Abstract]

#14

#12 OR #13

#15

#1 AND #2 AND #9 AND #14

OUTCOME: RENAL DISEASE

#16

((((("Kidney Diseases"[Mesh]) OR "Kidney"[Mesh]) OR "Proteinuria"[Mesh]) OR "Albumins"[Mesh]) OR "Creatinine"[Mesh] OR “glomerular filtration rate”[Mesh])

#17

nephropathy[Title/Abstract] OR kidney*[Title/Abstract] OR proteinuria[Title/Abstract] OR macroalbuminuria[Title/Abstract] OR renal[Title/Abstract] OR ckd[Title/Abstract] OR dkd[Title/Abstract] OR albumin[Title/Abstract] OR eskd[Title/Abstract] OR egfr[Title/Abstract] OR "glomerular filtration rate"[Title/Abstract] OR creatinine[Title/Abstract] OR acr[Title/Abstract]

#18

#16 OR #17

#19

#1 AND #2 AND #9 AND #18

RCT

#20

#1 AND #2 AND #8

SGLT2i

#21

"Diabetes Mellitus, Type 2"[Mesh] OR TD2[Title/Abstract] OR "Type 2 diabetes"[Title/Abstract] OR "Diabetes, type 2"[Title/Abstract

#22

"Sodium-Glucose Transporter 2 Inhibitors"[Mesh] Or "Sodium-Glucose Transporter 2"[Mesh]

#23

sglt2 OR sglt-2 OR sglt2-inhibitor* OR "sglt2 inhibitor*"[Title/Abstract] OR sglt2inhibitor* OR "sglt-2 inhibitor*"[Title/Abstract] OR sglt2i OR "sodium glucose transporter 2 inhibitor*"[Title/Abstract] OR "sodium glucose transporter-2 inhibitor*"[Title/Abstract] OR dapagliflozin OR empagliflozin OR ertugliflozin OR canagliflozin OR "sodium-glucose transporter 2"[Title/Abstract]

#24

#22 OR #23

#25

White[Title/Abstract] OR Caucasian[Title/Abstract] OR Asian[Title/Abstract] OR African[Title/Abstract] OR Ethnicity[Title/Abstract] OR Ethnic[Title/Abstract]

#26

Age[Title/Abstract] OR "Diabetes duration"[Title/Abstract] OR "BMI"[Title/Abstract] OR "Body Mass Index"[Title/Abstract] OR Adiposity[Title/Abstract] OR "Sex"[Title/Abstract] OR "clinical features"[Title/Abstract]

#27

"Insulin resistant"[Title/Abstract] OR "Insulin deficient"[Title/Abstract] OR "beta-cell function"[Title/Abstract] OR "c-peptide*"[Title/Abstract] OR "metabolite*"[Title/Abstract] OR "Metabolomic"[Title/Abstract] OR "Proteomic"[Title/Abstract] OR "Protein*"[Title/Abstract] OR "peptide*"[Title/Abstract]

#28

(Genotype[Title/Abstract] OR SNP[Title/Abstract] OR variant[Title/Abstract] OR GWAS[Title/Abstract] OR "Genome Wide Association Study"[Title/Abstract] OR Genetic[Title/Abstract] OR Epigenetic[Title/Abstract] OR Methylation[Title/Abstract]) OR ((("Genotype"[Mesh]) OR "Genome-Wide Association Study"[Mesh]) OR "Methylation"[Mesh])

#29

Precision[Title/Abstract] OR Prediction[Title/Abstract] OR Stratification[Title/Abstract] OR Stratified [Title/Abstract] OR Individualised[Title/Abstract] OR Individualisation[Title/Abstract] OR Personalized[Title/Abstract] OR Personalization[Title/Abstract] OR targeted[Title/Abstract]

#30

RCT OR randomis* OR randomiz*

#31

#25 OR #26 OR #27 OR #28 OR #29

Outcome: HbA1c reduction

#32

"hba1c reduction"[Title/Abstract] OR "hba1c change*"[Title/Abstract] OR "hba1c response"[Title/Abstract] OR "treatment response"[Title/Abstract] OR "glycaemic efficacy"[Title/Abstract] OR "glycemic efficacy"[Title/Abstract] OR fall in hba1c[Title/Abstract] OR change from baseline in hba1c[Title/Abstract] OR change in hba1c[Title/Abstract] OR changes in hba1c[Title/Abstract] OR slope of change[Title/Abstract] OR "hba1c outcome*"[Title/Abstract] OR outcome hba1c[Title/Abstract] OR "A1C change*"[Title/Abstract] OR "A1C reduction"[Title/Abstract] OR A1C response[Title/Abstract] OR A1C outcome*[Title/Abstract]

#33

#21 AND #24 AND #31 AND #32

Outcome: Cardiovascular disease

#34

((((((((("Cardiovascular Diseases"[Mesh]) OR "Myocardial Infarction"[Mesh]) OR "Myocardial Revascularization"[Mesh]) OR "Acute Coronary Syndrome"[Mesh]) OR "Myocardial Ischemia"[Mesh]) OR "Heart Failure"[Mesh]) OR "Angioplasty"[Mesh]) OR "Percutaneous Coronary Intervention"[Mesh]) OR "Stroke"[Mesh]) OR "Angina Pectoris"[Mesh])

#35

"cardiovascular disease"[Title/Abstract] OR "myocardial infarction"[Title/Abstract] OR revasculari*[Title/Abstract] OR "acute coronary syndrome"[Title/Abstract] OR "coronary heart disease"[Title/Abstract] OR "heart failure"[Title/Abstract] OR "cardiac failure"[Title/Abstract] OR angioplasty OR "percutaneous coronary intervention"[Title/Abstract] OR "cardiovascular mortality"[Title/Abstract] OR mace OR stroke OR angina OR "major adverse cardiovascular event*"[Title/Abstract] OR chd[Title/Abstract] OR acs[Title/Abstract] OR mi[Title/Abstract] OR hf[Title/Abstract] OR pci[Title/Abstract] OR cvd[Title/Abstract] OR sbp[Title/Abstract] OR dpb[Title/Abstract]

#36

#34 OR #35

#37

#21 AND #24 AND #31 AND #36

Outcome: Kidney disease

#38

((((("Kidney Diseases"[Mesh]) OR "Kidney"[Mesh]) OR "Proteinuria"[Mesh]) OR "Albumins"[Mesh]) OR "Creatinine"[Mesh] OR “glomerular filtration rate”[Mesh])

#39

nephropathy[Title/Abstract] OR kidney*[Title/Abstract] OR proteinuria[Title/Abstract] OR macroalbuminuria[Title/Abstract] OR renal[Title/Abstract] OR ckd[Title/Abstract] OR dkd[Title/Abstract] OR albumin[Title/Abstract] OR eskd[Title/Abstract] OR egfr[Title/Abstract] OR "glomerular filtration rate"[Title/Abstract] OR creatinine[Title/Abstract] OR acr[Title/Abstract]

#40

#38 OR #39

#41

#21 AND #24 AND #31 AND #40

RCT

#42

#21 AND #24 AND #30

Supplementary Table 1: Included studies for SGLT2i and Cardiovascular Disease

PMID	Author year	Study type	Data sources	Intervention (n)	Comparator (n)	Biomarkers examined	Significant interactions	Notes
Studies examining MACE outcomes								
27896705	Mahmoud 2017	Meta-analysis	EMPA-REG, CANVAS	Total = 22256		Sex	None	
30786725	Zelniker 2019	Meta-analysis	EMPA-REG, CANVAS, DECLARE	Total = 34322		CVD history	None	P=0.05 favoring greater SGLT-2 benefit in patients with CVD history
30424892	Zelniker 2019	Meta-analysis	EMPA-REG, CANVAS, DECLARE	19064	15258	Kidney function biomarkers (eGFR, creatinine, UACR); history of CVD; history of heart failure	None	
32993654	D'Andrea 2020	Meta-analysis	EMPA-REG, CANVAS, DECLARE	19064	15258	Age (at 65), sex, race (white, Black, Asian), CVD history, HF history, eGFR (at 60), HbA1c (at 8%), diabetes duration (at 10 years), blood pressure (at 130/80), BMI (at 30)	Greater SGLT benefit in patients with history of CVD	
32165164	Giugliano 2020	Meta-analysis	EMPA-REG, CANVAS, DECLARE	Total = 34323		Age, statin use	None	
31486272	Rådholm 2020	Meta-analysis	EMPA-REG, CANVAS, CREDENCE, DECLARE	NA	NA	Sex	None	
33031522	McGuire 2021	Meta-analysis	EMPA-REG, CANVAS, DECLARE, CREDENCE, VERTIS-CV	26765	20204	CVD history, HF history, eGFR (<60, 60-90, ≥90), ACR (normal, microalbuminuria, macroalbuminuria), HbA1c (<8.5, ≥8.5)	None	
34964748	Chang 2021	Meta-analysis	EMPA-REG, CANVAS, DECLARE, CREDENCE, VERTIS-CV, SCORED, SOLOIST-WHF	Total = 5873		Age (at 65), sex, race (white, other), diabetes duration (at 10 years), blood pressure (at 140/90), BMI (at 30), diabetes and CVD medication use	None	
34746739	Chun 2021	Meta-analysis	EMPA-REG, CANVAS, DECLARE, CREDENCE, VERTIS-CV, DAPA-HF, DAPA-CKD, EMPEROR-R, SCORED, SOLOIST-WHF	Total = 61821		eGFR (<45, 45-60, ≥60), ACR (normal, microalbuminuria, macroalbuminuria),	Greater SGLT-2 benefit with lower eGFR and higher ACR	

PMID	Author year	Study type	Data sources	Intervention (n)	Comparator (n)	Biomarkers examined	Significant interactions	Notes
33710721	Giugliano 2021	Meta-analysis	EMPA-REG, CANVAS, DECLARE, CREDENCE, VERTIS-CV, SCORED	Total = 65587		CVD history	None	
33986377	Uneda 2021	Meta-analysis	EMPA-REG, CANVAS, DECLARE, CREDENCE, VERTIS-CV	Total = 102728		BMI (at 30)	None	p-value for interaction not given
33707305	Lee 2021	Meta-analysis	EMPA-REG, CANVAS, VERTIS-CV	Total = 23556		Race (Asian vs. white)	None	
34780865	Kawai 2022	Meta-analysis	EMPA-REG, CANVAS, DECLARE, CREDENCE, VERTIS-CV, SCORED	31646	49560	ACR (normal, microalbuminuria, macroalbuminuria)	None	
34985519	Bhattarai 2022	Meta-analysis	EMPA-REG, CANVAS, DECLARE, CREDENCE, VERTIS-CV, CREDENCE DAPA-HF, DAPA-CKD, EMPEROR-R, SCORED, SOLOIST-WHF	39053	32500	Age (at 65), race (white vs. Asian/Black/other)	None	
28025462	Kaku 2017	RCT	EMPA-REG	4687	2333	Asian vs. other race/ethnicity	None	
29713728	Zinman 2018	RCT	EMPA-REG	4687	2513	Sex	None	
28904068	Wanner 2018	RCT	EMPA-REG	4687	2333	CKD (eGFR <60 and/or ACR ≥300)	None	
29777264	Verma 2018	RCT	EMPA-REG	4687	2333	History of CABG	None	
30882239	Furtado 2019	RCT	DECLARE	8582	8578	CVD history	Greater dapagliflozin benefit with history of CVD	
31530577	Neuen 2019	RCT	CANVAS	5740	4293	ACR (normal, microalbuminuria, macroalbuminuria)	None	
32744354	Wanner 2020	RCT	EMPA-REG	4687	2333	Categories of DKD by ACR and eGFR	None	
32795086	Bonaca 2020	RCT	DECLARE	8582	8578	PAD history	None	
32618884	Bohm 2020 (#331)	RCT	EMPA-REG	4687	2333	Decrease in SBP in response to treatment	None	
31820559	Bohm 2020 (#332)	RCT	EMPA-REG	4687	2333	AF history	None	
32227432	Verma 2020	RCT	EMPA-REG	4687	2333	TRS-HF-DM score	None	
32485734	Inzucchi 2020	RCT	EMPA-REG	4687	2333	Age, sex, BMI, HbA1c, eGFR, region, CVD risk factor goal attainment	None	
32030863	Verma 2019	RCT	EMPA-REG	4687	2333	Plasma uric acid (tertiles)	None	

PMID	Author year	Study type	Data sources	Intervention (n)	Comparator (n)	Biomarkers examined	Significant interactions	Notes
33307785	Langslet 2020	RCT	EMPA-REG	4623	2309	LDL cholesterol (5 categories)	None	
33004464	Neeland 2020	RCT	EMPA-REG	4687	2333	History of sleep apnea	None	
33611623	O'Donoghue 2021	RCT	DECLARE	8582	8578	Sex	None	
34427295	Oyama 2021	RCT	DECLARE	8570	8564	BMI, weight, adiposity markers	Greater dapagliflozin benefit in obese vs. non obese	MACE outcome included HHF
33851953	Zelniker 2021	RCT	DECLARE	8582	8578	eGFR, UACR	None	
32971190	Neuen 2021	RCT	CANVAS	5739	4292	KDIGO risk categories	None	
34415356	Sen 2021	RCT	CANVAS	2342	1181	Renal biomarkers: TNFR-1, TNFR-2, KIM-1	None	
34854308	Sen 2021	RCT	CANVAS	2357	1192	GDF-15 (tertiles)	None	
34908223	Kaku 2022	RCT	EMPA-REG	4623	2309	Asian vs. other race/ethnicity	None	Analyzed total MACE events (rather than time to event)
35115099	Vaduganathan 2022	RCT	CANVAS	2339	1164	Stress cardiac biomarkers: hs-cTnT, sST2, and IGFBP7	Greater canagliflozin benefit with higher levels of hs-cTnT and sST2, and more elevated biomarkers	
35120199	Oikonomou 2022	RCT	CANVAS	2886	1441	15-variable algorithm combining demographics and medical history	Algorithm predicted canagliflozin response	
28781064	Birkeland 2017	Observational	National registry data in Denmark, Sweden, Norway	22830	68490	Age, sex, history of renal disease, history of CVD, history of HF, background anti-hyperglycemic therapy, baseline antihypertensive therapy	Qualitative differences: stronger benefit of SGLT2i on MACE seen in older patients and those with pre-existing CVD.	
31902326	Raparelli 2020	Observational	Marketscan-Database	11774	89105	Sex	None	Composite outcome includes HF
34047459	Chan 2021	Observational	Chang Gung Memorial Hospital Research Database (Taiwan)	11769	na	Age, sex, BMI, weight, adiposity markers, HbA1c	Use of diuretic, prior stroke, older age, female sex, and lower BMI were associated with initial eGFR decline of >30% in those treated with SGLT2i; initial eGFR decline of >30% was	

PMID	Author year	Study type	Data sources	Intervention (n)	Comparator (n)	Biomarkers examined	Significant interactions	Notes
							associated with higher risk of new-onset atrial fibrillation and composite of MACE/HF in SGLT2i-treated individuals.	
33973690	Idris 2021	Observational	CPRD Aurum (England)	24438	24438 (DPP4i)	Age, sex, history of renal disease, history of CVD	None	
34570599	Patorno 2021	Observational	Medicare and 2 U.S. commercial claims data sets.	310417	246692	History of CVD	Greater benefit on MACE outcome for SGLT2i compared to GLP1RA in individuals with history of CVD than in those without history of CVD.	See below for HF outcome.
35132865	Htoo 2022	Observational	Medicare	24747	22596	History of CVD, history of heart failure, or both	Relative risk of MACE in SGLT2i-treated compared to GLP1RA-treated was higher in those without history of CVD and without history of HF; similar in those with history of CVD or history of HF; lower in those with history of CVD and history of HF.	See below for HF outcome.
Studies examining composite heart failure outcomes								
30424892	Zelniker 2019	Meta-analysis	EMPA-REG, CANVAS, DECLARE	19064	15258			
31486272	Rådholm 2020	Meta-analysis	EMPA-REG, CANVAS, CREDENCE, DECLARE	NA	NA	Sex	None	
33031522	McGuire 2021	Meta-analysis	EMPA-REG, CANVAS, DECLARE, CREDENCE, VERTIS-CV	26765	20204	CVD history, HF history, eGFR (<60, 60-90, ≥90), ACR (normal, microalbuminuria, macroalbuminuria), HbA1c (at 8.5%)	None	
34295696	Qiu 2021	Meta-analysis	EMPA-REG, DECLARE, VERTIS-CV, DAPA-HF, EMPEROR-R, SCORED, SOLOIST-WHF	NA	NA	Race (white, Black, Asian) CVD history, HF history, CKD history, NYHA HF class, LVEF, geographic region	Greater SGLT benefit in patients with NYHA HF Class II (vs. class III or IV) and in Black and	Subgroup analyses included subsets of CVOTs

PMID	Author year	Study type	Data sources	Intervention (n)	Comparator (n)	Biomarkers examined	Significant interactions	Notes
							Asian (vs. white) patients	
34964748	Chang 2021	Meta-analysis	EMPA-REG, CANVAS, DECLARE, CREDENCE, VERTIS-CV, SCORED, SOLOIST-WHF	Total = 5873		Age (at 65), sex, race (white, other), diabetes duration (at 10 years), blood pressure (at 140/90), BMI (at 30), diabetes and CVD medication use	None	
33609071	Bhatia 2021	Meta-analysis	EMPA-REG, CANVAS, DECLARE, CREDENCE, VERTIS-CV, DAPA-HF, DAPA-CKD, EMPEROR-R, SCORED, SOLOIST-WHF	Total = 71553		Mean age, sex, CVD history, HF history, CKD history, change in mediating biomarkers (HbA1c, blood pressure, body weight)	None	
33707305	Lee 2021	Meta-analysis	DAPA-HF, EMPEROR-R	Total = 23556		Race (Asian vs. white)	Greater SGLT-2 benefit in Asian vs. white	
34746739	Chun 2021	Meta-analysis	EMPA-REG, CANVAS, DECLARE, CREDENCE, VERTIS-CV, DAPA-HF, DAPA-CKD, EMPEROR-R, SCORED, SOLOIST-WHF	Total = 61821		eGFR (<45, 45-60, ≥60), ACR (normal, microalbuminuria, macroalbuminuria),	Greater SGLT-2 benefit with lower eGFR and higher ACR	
34985519	Bhattarai 2022	Meta-analysis	EMPA-REG, CANVAS, DECLARE, CREDENCE, VERTIS-CV, DAPA-HF, DAPA-CKD, EMPEROR-R, SCORED, SOLOIST-WHF	39053	32500	Age (at 65), race (white vs. Asian/Black/other)	None	
35020750	Li 2022	Meta-analysis	DECLARE, CREDENCE, VERTIS-CV, DAPA-HF, DAPA-CKD, EMPEROR-R, EMPEROR-P, SCORED, SOLOIST-WHF	14451	13372	eGFR (<30, 30-45, 45-60), HF history, CVD history	None	Population limited to CKD stage 3-4.
26819227	Fitchett 2016	RCT	EMPA-REG	4687	2333	Age (at 65), sex, race, HbA1c (at 8.5%), BMI (at 30), SBP (at 140/90), eGFR (<60, 60-90, ≥90), diabetes and CVD medication use	None	

PMID	Author year	Study type	Data sources	Intervention (n)	Comparator (n)	Biomarkers examined	Significant interactions	Notes
28025462	Kaku 2017	RCT	EMPA-REG	4687	2333	Asian vs. other race/ethnicity	None	
29713728	Zinman 2018	RCT	EMPA-REG	4687	2513	Sex	None	
29526832	Rådholm 2018	RCT	CANVAS	5795	4347	HF history	Greater canagliflozin benefit with history of HF	
28904068	Wanner 2018	RCT	EMPA-REG	4687	2333	CKD (eGFR <60 and/or ACR ≥300)	None	
29777264	Verma 2018	RCT	EMPA-REG	4687	2333	History of CABG	None	
30882239	Furtado 2019	RCT	DECLARE	8582	8578	CVD history	None	
30586757	Fitchett 2019	RCT	EMPA-REG	4687	2333	CVD history, CVD risk score	None	
31530577	Neuen 2019	RCT	CANVAS	5740	4293	ACR (normal, microalbuminuria, macroalbuminuria)	None	
31474116	Berg 2019	RCT	DECLARE	8582	8578	Risk score for HHF	None	
32744354	Wanner 2020	RCT	EMPA-REG	4687	2333	CKD categories by ACR and eGFR	None	
32795086	Bonaca 2020	RCT	DECLARE	8582	8578	PAD history	None	
33004464	Neeland 2020	RCT	EMPA-REG	4687	2333	History of sleep apnea	None	
32030863	Verma 2019	RCT	EMPA-REG	4687	2333	Plasma uric acid (tertiles)	None	
33307785	Langslet 2020	RCT	EMPA-REG	4623	2309	LDL cholesterol (5 categories)	None	
32618884	Bohm 2020 (#331)	RCT	EMPA-REG	4687	2333	Decrease in SBP in response to treatment	None	
31820559	Bohm 2020 (#332)	RCT	EMPA-REG	4687	2333	AF history	None	
32227432	Verma 2020	RCT	EMPA-REG	4687	2333	TRS-HF-DM score	None	
33611623	O'Donoghue 2021	RCT	DECLARE	8582	8578	Sex	None	
33950573	Ji 2021	RCT	EMPA-REG	4687	2333	3 BMI categories, stratified by Asian race	None	
34427295	Oyama 2021	RCT	DECLARE	8570	8564	BMI, weight, adiposity markers	Greater dapagliflozin benefit in obese vs. non-obese	
33851953	Zelniker 2021	RCT	DECLARE	8582	8578	eGFR, UACR	None	
32971190	Neuen 2021	RCT	CANVAS	5739	4292	KDIGO risk categories	None	
34364665	Savarese 2021	RCT	EMPA-REG	4675	2326	Cardiac ejection fraction prediction model	None	
34415356	Sen 2021	RCT	CANVAS	2342	1181	Renal biomarkers: TNFR-1, TNFR-2, KIM-1	None	
34854308	Sen 2021	RCT	CANVAS	2357	1192	GDF-15 (tertiles)	None	
34325887	Sharma 2021	RCT	EMPA-REG	Total = 6639		Latent class analysis by	None	

PMID	Author year	Study type	Data sources	Intervention (n)	Comparator (n)	Biomarkers examined	Significant interactions	Notes
						demographics and clinical features		
34908223	Kaku 2022	RCT	EMPA-REG	4623	2309	Asian vs. other race/ethnicity	None	Analyzed total HF events (rather than time to event)
35115099	Vaduganathan 2022	RCT	CANVAS	2339	1164	Stress cardiac biomarkers: hs-cTnT, sST2, and IGFBP7	None	
29935543	Kim 2018	Observational	Korean Health Insurance Review and Assessment Service	59479	59479 (DPP4i)	History of CVD	Relative benefit in reducing HF hospitalization with SGLT2i vs DPP4i treatment seen more quickly in those with CVD history than in those without	
31902326	Raparelli 2020	Observational	Marketscan-Database	11774	89105 (SU)	Sex	None	Composite outcome includes HF
33599357	Becher 2021	Observational	Swedish heart failure registry	361	1083	Kidney function biomarkers (eGFR, creatinine, UACR), background anti-hyperglycemic therapy, heart failure type (HFpEF, HFmrEF, HFrEF)	No interactions observed by HF subtype, concomitant metformin treatment, or eGFR	
33973690	Idris 2021	Observational	CPRD Aurum (England)	24438	24438 (DPP4i)	Age, sex, history of renal disease, history of CVD	None	
34570599	Patorno 2021	Observational	Medicare and 2 U.S. commercial claims data sets.	310417 (SGLT2i)	246692 (GLP1RA)	History of CVD	No difference in relative benefit of SGLT2i compared to GLP1RA in individuals with or without history of CVD.	See above for MACE outcome.
35132865	Htoo 2022	Observational	Medicare	24747 (SGLT2i)	22596 (GLP1RA)	History of CVD, history of heart failure, or both	Relative reduction in HF hospitalization was greater for SGLT2i compared to GLP1RA in those with history of HF but not of CVD.	See above for MACE outcome.

Abbreviations: atrial fibrillation (AF), coronary artery bypass grafting (CABG), diabetic kidney disease (DKD), growth differentiation factor-15 (GDF-15), heart failure (HF), heart failure with mid-range ejection fraction (HFmrEF), heart failure with preserved ejection fraction (HFpEF), heart failure with

reduced ejection fraction (HFrEF), hospitalisation for heart failure (HHF), high-sensitivity cardiac troponin T (hs-cTNT), insulin-like growth factor binding protein 7 (IGFBP7), Kidney Disease Improving Global Outcomes (KDIGO), kidney injury molecule-1 (KIM-1), left ventricular ejection fraction (LVEF), New York Heart Association (NYHA), peripheral arterial disease (PAD), soluble suppression of tumorigenesis-2 (sST2), tumor necrosis factor receptor 1 (TNFR-1), tumor necrosis factor receptor 2 (TNFR-2).

Supplementary Table 2: Included studies for GLP1RA and Cardiovascular Disease

PMID	Author (year)	Study type	Data sources	Intervention (n)	Comparator (n)	Biomarkers examined	Significant interactions	Notes
30566004	Verma 2018	RCT	LEADER	4668	4672	History of CVD	Liraglutide reduced risk of CV outcomes in individuals with history of MI/stroke, but not in those without history.	
32744418	Verma 2020	RCT	LEADER; SUSTAIN 6	6316	6321	Adiposity biomarkers	Semaglutide more efficacious with higher baseline BMI.	Only seen in SUSTAIN 6, LEADER showed no GLP1RA/biomarker interaction.
30851070	Verma 2019	RCT	LEADER; SUSTAIN 6	NA	NA	Diabetes duration	None	
32643857	Verma 2020	RCT	LEADER; SUSTAIN 6	NA	NA	Microvascular disease	None	
33537745	Riddle 2021	RCT	REWIND	4949	4952	Age	None	
32618386	Mosenzon O 2020	RCT	LEADER	4668	4672	UACR and eGFR strata	None	
30371301	Mentz 2018	RCT	EXSCEL	7356	7356	Cardiovascular risk score	None	
32164886	Marso 2020	RCT	LEADER	4668	4672	History of heart failiure	None	
30566006	Mann 2018	RCT	LEADER	4668	4672	Kidney function biomarkers	None	
31167654	Leiter 2019	RCT	SUSTAIN 6	1648	1649	Age; Sex; History of CVD	None	
32372454	Leiter 2020	RCT	LEADER; SUSTAIN 6	NA	NA	Blood pressure strata	None	
30508430	Gilbert 2019	RCT	LEADER	4668	4672	Age	None	
31542942	Fudim 2019	RCT	EXSCEL	7356	7396	History of heart failure	Exenatide had attenuated efficacy in individuals with HF at baseline.	
33905751	Barbery 2021	RCT	EXSCEL	7356	7396	Sex; Ethnicity; History of CVD; History of heart failure; Smoking status	Individuals on exenatide enrolled in Latin America experienced lower risk of ACS or coronary revascularisation. Exenatide had no differential effect in individuals with or without CVD history.	
31752517	Badjatiya 2019	RCT	EXSCEL	7355	7396	History of CVD	None	Exenatide had no effect on CVD risk in individuals with or without PAD.
29935211	Wang 2018	Meta-analysis	LEADER; ELIXA;	16706	16751	Sex; Ethnicity; Adiposity biomarkers;	CV benefits associated with GLP-1RA use was	

PMID	Author (year)	Study type	Data sources	Intervention (n)	Comparator (n)	Biomarkers examined	Significant interactions	Notes
			SUSTAIN 6; EXSCEL;			Kidney function biomarkers; History of CVD; Background anti-hyperglycaemic therapy	only observed in male, black, Asian, or obese patients.	
34144086	Tsapas 2021	Meta-analysis	HARMONY OUTCOMES; REWIND; EXSCEL; LEADER	NA	NA	Background treatment with metformin	None	
32077924	Marsico 2020	Meta-analysis	ELIXA; LEADER; SUSTAIN 6; EXSCEL; HARMONY; REWIND; PIONEER 6	27977	28027	History of CVD with or without prior MI; History of CVD; Cardiovascular risk factors	None	
31903692	Husain 2020	Pooled RCT	SUSTAIN 6; PIONEER 6	3239	3241	History of CVD; History of CV outcome	Semaglutide less efficacious in individuals with prior HF.	Semaglutide showed consistent effects on MACE versus comparators across CV risk categories.
32998732	Husain 2020	Pooled RCT	SUSTAIN 6; PIONEER 6	10508	7137	Cardiovascular risk score	Relative risk reduction was largest in the low CV risk score group; the largest absolute risk reduction was in the intermediate/high CV risk score group.	
32734559	He 2020	Meta-analysis	LEADER; SUSTAIN 6; EXSCEL; HARMONY OUTCOMES; REWIND; PIONEER 6	24583	24633	Age; Sex; Diabetes duration; Adiposity biomarkers; Glycaemic biomarkers; Kidney function biomarkers; History of CVD; Geographic location	None	Slight trend observed suggesting established CVD may have increased benefit to GLP-1RAs.
31373167	Giugliano 2019	Meta-analysis	REWIND; PIONEER 6	NA	NA	History of CVD	None	
34780865	Kawai 2022	Meta-analysis	ELIXA; LEADER; REWIND	24362	15936	Kidney function biomarkers; Albuminuria status	None	
31595657	Mannucci 2019	Meta-analysis	ELIXA; LEADER; SUSTAIN 6; EXSCEL; HARMONY; REWIND; PIONEER-6	27977	28027	Age; Sex; Adiposity biomarkers; Kidney function biomarkers; History of CVD; Geographic location	None	

PMID	Author (year)	Study type	Data sources	Intervention (n)	Comparator (n)	Biomarkers examined	Significant interactions	Notes
30786725	Zelniker 2019	Meta-analysis	ELIXA; LEADER; SUSTAIN-6; EXSCEL; HARMONY; EMPA-REG OUTCOME	NA	NA	History of cardiovascular disease	GLP1RAs only reduced MACE in patients with established CVD compared to those without;	
33986377	Uneda 2021	Meta-analysis	ELIXA; EXSCEL; LEADER; HARMONY Outcomes; PIONEER 6; REWIND; SUSTAIN 6	NA	NA	Adiposity biomarkers	None	
32142999	Singh 2020	Meta-analysis	ELIXA; LEADER; SUSTAIN 6; EXSCEL; HARMONY; REWIND; PIONEER 6	NA	NA	Sex	None	
NA	Qiu 2020	Meta-analysis	ELIXA; LEADER; SUSTAIN-6; EXSCEL; HARMONY OUTCOMES; REWIND; PIONEER 6	NA	NA	Sex; Diabetes duration; History of CVD; Background anti-hyperglycaemic therapy	None	
33707305	Lee 2021	Meta-analysis	LEADER; SUSTAIN 6; EXSCEL; HARMONY OUTCOMES; REWIND; PIONEER 6	NA	NA	Ethnicity	GLP1RA use in Asians with T2D associated with reduced CVD outcome risk.	
32993654	D'Andrea 2020	Meta-analysis	ELIXA; LEADER; SUSTAIN 6; EXSCEL; HARMONY; REWIND; PIONEER 6	27681	27757	Age; Sex; Diabetes duration; Ethnicity; Adiposity biomarkers; Glycaemic biomarkers; Kidney function biomarkers; History of CVD; History of heart failure; Blood pressure	GLP1RAs only showed CVD protective effect in individuals with prior CVD.	Trend towards greater cardiovascular protection in individuals with uncontrolled diabetes. Uncontrolled hypertension, obesity, gender, age, and race did not modify the effect of GLP1RAs.
32534570	Yang 2020	Observational	National Health Insurance Research Database of Taiwan	5089	5089	Age; Sex; Diabetes duration; History of CVD; Microvascular disease	GLP1RA versus DPP-4i yielded greater cardiovascular benefit in those without established CVD versus those with established CVD.	
35254430	Chen 2022	Observational	National Health Insurance Research	701	26578	Age; Sex; Cardiovascular outcome; ACE inhibitor use; End stage	Lower risk of mortality associated with use of GLP1RAs compared with	

PMID	Author (year)	Study type	Data sources	Intervention (n)	Comparator (n)	Biomarkers examined	Significant interactions	Notes
			Database of Taiwan			kidney disease status	DDP-4 inhibitors among patients with cerebrovascular disease than those without.	
34570599	Patorno 2021	Observational	Medicare and 2 U.S. commercial claims data sets.	246692	310417	History of CVD	Greater cardiovascular benefit in individuals with CVD compared to without.	
31902326	Raparelli 2020	Observational	Marketscan-Database	14697	152557	Sex	Greater cardiovascular effectiveness in women.	
35132865	Htoo 2022	Observational	Medicare	22596	24747	History of CVD; History of Cardiovascular Outcome	Atherosclerotic CVD events were less frequent with GLP1RA in those without prior CVD or HF.	

Abbreviations**:** angiotensin-converting-enzyme (ACE), myocardial infarction (MI).

Supplementary Table 3: Studies not included for GLP1RA and Cardiovascular and reasons for non-inclusion

Author year	Reason for not including
Bohm 2020 (Heart failure and renal outcomes according to baseline and achieved blood pressure in patients with type 2 diabetes: results from EMPA-REG OUTCOME)	Did not report statistical test of interaction
Chan 2021	No control group
Idris 2021	Did not report statistical test of interaction
Kawai 2022	Did not report statistical test of interaction
Lin 2021	Did not report statistical test of interaction
Nunoi 2019	Did not report statistical test of interaction
Xie 2020	Did not report statistical test of interaction
Zhou 2019	Machine learning model identified combinations of biomarkers rather than individual ones

Supplementary Table 4: Included studies for SGLT2i and Renal Disease

PMID	Author year	Study type	Data sources/trials	N (intervention)	N (comparator)	Biomarkers examined	Significant interactions	Notes
Studies examining eGFR changes / CKD progression / composite outcomes of these with or without ACR changes								
31506585	Bae 2019	Meta-analysis	48 studies	34,661	23,504	eGFR (continuous)	None	Non-significant greater SGLT-2 benefit with higher eGFR
34964748	Chang 2021	Meta-analysis	EMPA-REG, CANVAS, DECLARE, CREDENCE (VERTIS-CV, SCORED, SOLOIST-WHF not used for renal outcomes)	(58,783 in intervention + comparator arm, not given separately)	(58,783 in intervention + comparator arm, not given separately)	Age (<65, ≥65 years), sex, race (white, other), diabetes duration (<10, ≥10 years), blood pressure (SBP≥140 or DBP≥90, SBP<140 and DBP<90 mmHg) , BMI (<30, ≥30 kg/m^2), diabetes and CVD medication use	None	
34746739	Chun 2021	Meta-analysis	EMPA-REG, CANVAS, DECLARE, CREDENCE, DAPA-CKD, SCORED (DAPA-HF, VERTIS-CV, EMPEROR-R, SOLOIST-WHF not used for renal outcomes)	(61,821 in intervention + comparator arm, not given separately)	(61,821 in intervention + comparator arm, not given separately)	eGFR (<45, 45-<60, ≥60), ACR (<30 [normoalbuminuria], 30-300 [microalbuminuria], >300 [macroalbuminuria])	None	
33710721	Giugliano 2021	Meta-analysis	EMPA-REG, CANVAS, DECLARE, CREDENCE, VERTIS-CV, SCORED, DAPA-CKD, EMPEROR-R	(65,587 in intervention + comparator arm, not given separately)	(65,587 in intervention + comparator arm, not given separately)	CVD history	None	
33031522	McGuire 2021	Meta-analysis	EMPA-REG, CANVAS, DECLARE, CREDENCE, VERTIS-CV	(46,969 in intervention + comparator arm, not given separately)	(46,969 in intervention + comparator arm, not given separately)	CVD history, HF history, ACR (<30 [normoalbuminuria], 30-300 [microalbuminuria], >300 [macroalbuminuria])	None	
31495651	Neuen 2019 (SGLT2 inhibitors for the prevention of kidney failure in patients with type 2 diabetes: a systematic review and meta-analysis)	Meta-analysis	EMPA-REG, CANVAS, DECLARE, CREDENCE	(38,723 in intervention + comparator arm, not given separately)	(38,723 in intervention + comparator arm, not given separately)	CVD medication (RAS blockade) use, eGFR (<45, 45-<60, 60-<90, ≥90), ACR (<30 [normoalbuminuria], 30-300 [microalbuminuria], >300 [macroalbuminuria])	None	Non-significant trend for greater SGLT2i effect in those with higher baseline eGFR

PMID	Author year	Study type	Data sources/trials	N (intervention)	N (comparator)	Biomarkers examined	Significant interactions	Notes
30424892	Zelniker 2019	Meta-analysis	EMPA-REG, CANVAS, DECLARE	19,064	15,258	CVD status (ASCVD vs multiple risk factors), eGFR (<60, 60-<90, ≥90)	None for CVD status; greater SGLT-2 benefit in patients with higher eGFR	
33214158	Bakris 2020	RCT	CREDENCE	2,202	2,199	eGFR (<30, ≥30)	None	
31820559	Bohm 2020 (Efficacy of empagliflozin on heart failure and renal outcomes in patients with atrial fibrillation: data from the EMPA-REG OUTCOME trial)	RCT	EMPA-REG	4,687	2,333	AF history	None	
32795086	Bonaca 2020	RCT	DECLARE	8,582	8,578	PAD history	None	
33158949	Januzzi 2021	RCT	CANVAS	2,384	1,193	IGFBP7 (quartiles)	None	
33619120	Jardine 2021	RCT	CREDENCE	2,202	2,199	ACR (all in macroalbuminuria range: ≤1000, >1000-<3000, ≥3000)	None	
33950573	Ji 2021	RCT	EMPA-REG	4,687	2,333	3 BMI categories (different for Asian vs non-Asian), stratified by Asian race	None	
30412655	Kadowaki 2019	RCT	EMPA-REG	4,687	2,333	Race (Asian, other)	None	
34233928	Mosenzon 2021	RCT	DECLARE	8,582	8,578	ACR (≤15, 15<30, 30-300 [microalbuminuria], >300 [macroalbuminuria])	None for renal outcome; greater SGLT-2 benefit with higher ACR for cardiorenal outcome (micro- and macroalbuminuria have greater benefit than other categories)	
33004464	Neeland 2020	RCT	EMPA-REG	4,687	2,333	History of sleep apnea	None	Very small numbers with sleep apnea
31530577	Neuen 2019 (Effect of Canagliflozin on Renal and Cardiovascular Outcomes across Different Levels of Albuminuria: Data from the CANVAS Program)	RCT	CANVAS	5,740	4,293	ACR (<30 [normoalbuminuria], 30-300 [microalbuminuria], >300 [macroalbuminuria])	Greater SGLT-2 benefit with higher ACR for preventing eGFR decline. Also heterogeneity in composite renal outcome by baseline UACR (no benefit of SGLT-2 in microalbuminuria but large benefit for normo- or macroalbuminuria)	
32971190	Neuen 2021	RCT	CANVAS	(10,031 in intervention + comparator	(10,031 in intervention + comparator	KDIGO risk categories	None	

PMID	Author year	Study type	Data sources/trials	N (intervention)	N (comparator)	Biomarkers examined	Significant interactions	Notes
				arm, not given separately)	arm, not given separately)	(composite of eGFR and ACR)		
33611623	O'Donoghue 2021	RCT	DECLARE	8,582	8,578	Sex	None	
34427295	Oyama 2021	RCT	DECLARE	8,570	8,564	BMI (18.5 to <25, 25 to <30, 30 to <35, 35 to <40, ≥40 kg/m^2)	None	
34854308	Sen 2021 (Association Between Circulating GDF-15 and Cardio-Renal Outcomes and Effect of Canagliflozin: Results from the CANVAS Trial)	RCT	CANVAS	2,357	1,192	GDF-15 (tertiles)	None	
34415356	Sen 2021 (Effects of the SGLT2 inhibitor canagliflozin on plasma biomarkers TNFR-1, TNFR-2 and KIM-1 in the CANVAS trial)	RCT	CANVAS	2,342	1,181	Renal biomarkers: TNFR-1, TNFR-2, KIM-1 (tertiles and continuous)	None	Greater SGLT-2 benefit in lowest TNFR-2 tertile (although few events), but non-significant when TNFR-2 treated as a continuous variable
35115099	Vaduganathan 2022	RCT	CANVAS	2,339 (fewer in total for sST2 and more for IGFBP7)	1,164 (fewer in total for sST2 and more for IGFBP7)	Stress cardiac biomarkers: hs-cTnT (<14, ≥14 pg/mL and continuous), sST2 (<35, ≥35 ng/mL and continuous), and IGFBP7 (<96.5, ≥96.5 ng/mL and continuous)	None	
29777264	Verma 2018	RCT	EMPA-REG	4,687	2,333	History of CABG	None	
32030863	Verma 2020	RCT	EMPA-REG	4,686	2,326	Plasma uric acid (tertiles)	None	
32744354	Wanner 2020	RCT	EMPA-REG	4,687	2,333	CKD (composite of eGFR <60 and/or ACR ≥300)	None	
33118320	Koh 2021	Observational	CVD-REAL 3 Korea (insurance database)	45,016	45,016	Age (<65, ≥65 years), sex, BMI (<25, ≥25 kg/m^2), abdominal obesity, diabetic retinopathy, hypertension, CVD history, eGFR (<60, 60-<90, ≥90), proteinuria, eGFR + proteinurea(<60 + absent, ≥60 + absent, <60 + present, ≥60 + present)	Greater SGLT-2 benefit with higher BMI, with abdominal obesity, and with lower eGFR (<90 vs ≥90)	

PMID	Author year	Study type	Data sources/trials	N (intervention)	N (comparator)	Biomarkers examined	Significant interactions	Notes
34593566	Nagasu 2021	Observational	Japan Chronic Kidney Disease Database (registry)	1,033	1,033	Age (<65, ≥65 years), eGFR (<60, ≥60), rapid decline in eGFR before initiating treatment, proteinuria, ACE inhibitor/ARB use	Greater SGLT-2 benefit on eGFR when no rapid eGFR decline and when ACE inhibitor/ARB not used	No interaction for composite renal outcome of eGFR decline and ESRD
Studies examining ACR changes								
31506585	Bae 2019	Meta-analysis	48 studies	34,661	23,504	ACR (continuous)	None	Non-significant greater SGLT-2 benefit with higher ACR
33158949	Januzzi 2021	RCT	CANVAS	2,384	1,193	IGFBP7 (quartiles)	Greater SGLT-2 benefit on 'first progression of albuminuria' outcome with higher ICFBP7	
30412655	Kadowaki 2019	RCT	EMPA-REG	4,687	2,333	Race (Asian, other)	None	

Abbreviations: angiotensin-converting-enzyme (ACE), atrial fibrillation (AF), angiotensin receptor blocker (ARB), coronary artery bypass grafting (CABG), end stage renal disease (ESRD), growth differentiation factor-15 (GDF-15), high-sensitivity cardiac troponin T (hs-cTNT), insulin-like growth factor binding protein 7 (IGFBP7), Kidney Disease Improving Global Outcomes (KDIGO), kidney injury molecule-1 (KIM-1), renin–angiotensin system (RAS), soluble suppression of tumorigenesis-2 (sST2), tumor necrosis factor receptor 1 (TNFR-1), tumor necrosis factor receptor 2 (TNFR-2).

Supplementary Table 5: Included studies for GLP1RA and Renal Disease

PMID	Author year	Study type	Data sources/trials	Intervention (n)	Comparator (n)	Biomarkers examined	Significant interactions	Notes
Studies examining eGFR changes / CKD progression / composite outcomes of these with or without ACR changes								
32372454	Leiter 2020	RCT	LEADER, SUSTAIN 6 (analysed separately)	(9,340 [LEADER], 3,297 [SUSTAIN 6] in intervention + comparator arm, not given separately)	(9,340 [LEADER], 3,297 [SUSTAIN 6] in intervention + comparator arm, not given separately)	Blood pressure (<120/80 [normal], SBP 120-129 and DBP<80 [elevated], SBP 130-139 or DBP 80-89 [stage 1 hypertension], SBP≥140 or DBP≥90 mmHg [stage 2 hypertension])	None	
32164886	Marso 2020	RCT	LEADER	4,668	4,672	HF history	None	
32618386	Mosenzon 2020	RCT	LEADER	4,668	4,672	eGFR (<30, 30-<45, 45-<60, 60-<90, ≥90), ACR (0, >0-<15, 15-<30, 30-<100, 100-<300 [microalbuminuria], ≥300 [macroalbuminuria])	None	
30292589	Muskiet 2018	RCT	ELIXA	2,984	2,994	ACR (<30 [normoalbuminuria], 30-300 [microalbuminuria], >300 [macroalbuminuria])	None	
33537745	Riddle 2021	RCT	REWIND	4,949	4,952	Age (at 65)	None	
34903039	Shaman 2022	Pooled RCT	LEADER, SUSTAIN 6	6,316	6,321	eGFR (<30, 30-<60, 60-<90, ≥90)	Greater GLP1RA benefit with eGFR 30-60 (other categories: no treatment effect)	
32803900	vanderAart-vanderBeek 2020	RCT	EXSCEL	6,906	6,920	Blood pressure (SBP≥140, SBP<140 mmHg), BMI (<30, ≥30 kg/m^2), eGFR (<60, ≥60), CVD history, CVD medication (RAASi)	Greater GLP1RA benefit with lower BMI	Also looked at ACR (≤30, >30-100, >100-200, >200): greater GLP1RA benefit on eGFR decline with higher ACR although no statistical test for interaction
30851070	Verma 2019	RCT	LEADER, SUSTAIN 6 (analysed separately)	(9,321 [LEADER], 3,297 [SUSTAIN 6] in intervention + comparator arm, not given separately)	(9,321 [LEADER], 3,297 [SUSTAIN 6] in intervention + comparator arm, not given separately)	Diabetes duration (<5, 5-<15, 15-<25, ≥25 years)	None	
32744418	Verma 2020	RCT	LEADER, SUSTAIN 6 (analysed separately)	4,668 (LEADER), 1,648 (SUSTAIN 6)	4,672 (LEADER), 1,649 (SUSTAIN 6)	BMI (<25, 25-<30, 30-<35, ≥35 kg/m^2)	None	

PMID	Author year	Study type	Data sources/trials	Intervention (n)	Comparator (n)	Biomarkers examined	Significant interactions	Notes
Studies examining ACR changes								
30292589	Muskiet 2018	RCT	ELIXA	2,984	2,994	ACR (<30 [normoalbuminuria], 30-300 [microalbuminuria], >300 [macroalbuminuria])	Greater GLP1 benefit with higher ACR (no treatment effect with normoalbuminuria but strong reduction in proteinuria in those with micro and macroalbumunuria)	
34903039	Shaman 2022	Pooled RCT	LEADER, SUSTAIN 6	6,316	6,321	ACR (<30 [normoalbuminuria], 30-300 [microalbuminuria], >300 [macroalbuminuria])	Greater GLP1 benefit with microalbuminuria than normo- or macroalbuminuria	
32803900	vanderAart-vanderBeek 2020	RCT	EXSCEL	6,906	6,920	Blood pressure (SBP≥140, SBP<140 mmHg), BMI (<30, ≥30 kg/m^2), eGFR (<60, ≥60), CVD history, CVD medication (RAASi)	None	

Abbreviations: renin-angiotensin-aldosterone system inhibitors (RAASi).

Supplementary Table 6: Studies not included for SGLT2 and Renal Disease or GLP1RA and Renal Disease, and reasons for non-inclusion

Author year	Reason for not including
Kawai 2022	Did not report statistical test of interaction
Xie 2020	Did not report statistical test of interaction

Supplementary Table 7: Included studies for SGLT2i and Glycaemic Outcomes

PMID	Author (year)	Study type	Data Source	Intervention (n)	Comparator (n)	Biomarkers examined	Significant interactions	Notes
32821142	Scheen (2020)	Meta-Analysis	7 RCTs of Asian patients; 16 RCTs of Non-Asian patients	1164	1088	Ethnicity	None	Greater reduction in HbA1c for Asians vs Non-Asians
29029369	Cai (2018)	Meta-Analysis	17 RCTs of Asian patients; 39 RCTs of Non-Asian patients	5679	4170	Ethnicity	None	
35314533	Wang (2022)	Meta-Analysis	Adhimadhyam (2018); Amos (2016); Cefalu (2015); Chilton: cohort 1 (2015); Chilton: cohort2 (2015); Gautam (2017); Johnson (2017); Kobayashi (2019); Kohler (2016); Leiter (2014); Maegawa (2018); Osonoi (2018); Shiba (2017); Sinclair (2014)	287	921	Age	None	SGLT2i benefitted patients aged <65 years for HbA1c reduction
28583425	Wilding (2017)	Observational		5825	0	Background AHA	None	
27822077	Scheerer (2016)	Observational		1169	0	Age; Sex; History of CVD; Background AHA	Higher HbA1c at baseline was significantly associated with greater HbA1c reduction	
32744394	Montvida (2020)	Observational	Centricity Electronic Medical Records Database (USA)	82694	0	Ethnicity	Black patients less likely to achieve glycaemic control (HbA1c < 7.5% or 1% reduction) at 18 months than white patients	White and black patients had similar HbA1c reduction at 6 months
31369642	Cho (2019)	Observational	Korean outpatient clinic	374	0	Age; Sex; Diabetes duration; Adiposity markers; Glycaemic biomarker	Hba1c reductions greater in younger patients (≤50 years) compared to older patients (>60 years)	HbA1c reduction significantly greater for patients with higher baseline HbA1c; no difference in glycaemic efficacy for BMI subgroups
33240585	Chen (2020)	Observational	Multicenter observational patient data (Taiwan)	1197	0	Age; Sex; Adiposity markers; Glycaemic biomarker;	Patients with higher baseline HbA1c showed greater	

						Background AHA	reduction in HbA1c at 6 months	
28829163	Brown (2017)	Observational	Canadian diabetes registry	1520	0	Age; Sex; Diabetes duration; Adiposity markers; Glycaemic biomarker; History of Renal Disease; CV biomarkers; History of hypertension	Greater reductions in HbA1c seen in patients taking dapagliflozin with higher baseline HbA1c, shorter duration of diabetes, or male sex	
32424798	Strain (2020)	Observational		490	6680	Background AHA	SGLT2is associated with greater HbA1c reduction in younger patients	Males, lower baseline BMI, and higher baseline DBP also associated with better response for SGLT2is (NS)
30688052	Lee (2019)	Observational	Korean outpatient clinic	804	0	Adiposity markers; Background AHA	Higher HbA1c at baseline was significantly associated with greater HbA1c reduction	
31050099	Zhou (2019)	Observational	Medical Data Vision Database (Japan)	990	4257	Diabetes duration; Kidney function biomarkers; CV biomarkers; Glycaemic biomarkers; Use of anti-thrombotic agents	None	
30815552	DeFronzo (2017)	Pooled RCT	EMPA-REG MONO; EMPA-REG H2H-SU	1213	1003	Glycaemic markers	Patients with higher baseline HbA1c on empagliflozin showed significantly greater reductions in HbA1c than those on sitagliptin or glimepiride	

28860019	Cherney (2018)	Pooled RCT	Haring (2013); Haring (2014); Kovacs (2014); Roden (2013); Barnett (2014)	1142	1144	Kidney function markers	None	HbA1c reduction greater for higher baseline eGFR subgroups (≥60 ml/min/1.72m^2)
24742013	Sinclair (2014)	Pooled RCT	Stenlof (2013); Lavalle-Gonzalez (2013); Wilding (2013)	1667	646	Age	None	
25059406	Yamout (2014)	Pooled RCT	Stenlof (2014); Yale (2013); Bode (2013); Neal (2013)	703	382	Kidney function markers	None	LS mean reductions in HbA1c for patients with stage 3a CKD was greater than those with stage 3b CKD; no formal statistical testing performed performed
26600115	Matthews (2016)	Pooled RCT	Stenlof (2013); Lavalle-Gonzalez (2013); Wilding (2013); Forst (2014); Schernthaner (2013)	1316	1156	Glycaemic markers; HOMA2-%B and HOMA2-%S markers	None	Investigated baseline beta-cell function and insulin sensitivity via HOMA2-%B/S tertiles
32324082	Liu (2020)	Pooled RCT		1029	515	Ethnicity	None	
27052454	Gilbert (2016)	Pooled RCT	Stenlof (2013); Lavalle-Gonzalez (2013); Wilding (2013); Forst (2013); Neal (2015); Fulcher (2015)	2763	1176	Age; Sex; Adiposity markers; Kidney function markers	None	
26373629	Blonde (2015)	Pooled RCT	NCT0108183 NCT01106677NCT01106625NCT01106690	1667	646	Age; Sex	None	
31933292	Parigi (2019)	Pooled RCT	Lewin (2015); DeFronzo (2015)	1080	261	Age; Sex; Diabetes duration; Ethnicity; Adiposity markers; Glycaemic markers; Kidney function biomarkers	None	
32700421	Pratley (2020)	Pooled RCT	VERTIS-MONO; VERTIS-MET; VERTIS-SITA2; VERTIS-SU; VERTIS-SITA; VERTIS-FACTORIAL;VERTIS-RENAL	2533	1072	Age	None	No formal statistical testing performed
33004464	Neeland (2020)	RCT	EMPA-REG OUTCOME	4687	2333	Obstructive Sleep Apnea	None	
29573139	Frias (2018)	RCT	DURATION-8	233	462	Age; Sex; Diabetes duration; Ethnicity; Adiposity markers;	None	NS association for age and baseline eGFR; dapagliflozin + exenatide resulted in

						Glycaemic markers; Kidney function biomarkers		larger HbA1c reduction than either AHA alone
35233908	Cherney (2022)	RCT	VERTIS-CV	5499	2747	Kidney function biomarkers	HbA1c reductions lowest in patients with high/very high risk of CKD	
28904068	Wanner (2018)	RCT	EMPA-REG OUTCOME	4687	2333	Kidney function biomarkers	None	
33084149	Inzucchi (2021)	RCT	EMPA-REG MET	430	207	Adiposity markers; Glycaemic markers; Systolic Blood Pressure	Higher HbA1c at baseline was significantly associated with greater HbA1c reduction	

Abbreviations: anti-hyperglycaemic agents (AHA), Homeostasis Model Assessment 2 - %B (beta cell; HOMA2-%B), Homeostasis Model Assessment 2 - %S (insulin sensitivity; HOMA2-%S).

Supplementary Table 8: Included studies for GLP1RA and Glycaemic Outcomes

PMID	Author (year)	Study type	Data Source	Intervention (n)	Comparator (n)	Biomarkers examined	Significant interactions	Notes
33452595	Yang 2021	RCT	ChiCTR-IPR-15006558	190	0	Serum FGF21	High baseline FGF21 levels are associated with poor glycaemic response to exenatide in patients with type 2 diabetes.	
27027802	Wolffenbuttel 2016	RCT	NCT00960661; NCT00765817	452	434	Adiposity markers	None	
35311356	Geng 2022	RCT	CONFIDENCE	100	0	Genetics	SNP rs163184 in the gene *KCNQ1* was associated with reduced glycaemic response to exenatide in T2DM patients.	
25619391	Feng 2015	RCT	NA	328	0	Age; Adiposity biomarkers; Glycaemic biomarkers; Kidney function biomarkers; Fasting c peptide; Fasting insulin	Patients with high insulin-secreting ability, hyperglucagonemia, and short-duration diabetes may obtain better glycaemic control with liraglutide.	
27161178	Boustani 2016	RCT	AWARD	3136	2035	Age	None	
29573139	Frias 2018	RCT	DURATION 8	461	233	Age; Sex; Diabetes duration; Ethnicity; Adiposity biomarkers; Glycaemic biomarkers; Kidney function biomarkers	Treatment-by-subgroup interaction was observed for HbA1c change by baseline age subgroup, and HbA1c reductions were greater for patients with higher eGFR. Baseline BMI, T2D duration, sex, race, and ethnicity did not affect HbA1c reductions.	Small group sizes for >65 years and race make those results difficult to conclusively interpret.
32277401	Yu 2020	Meta-analysis	AWARD-CHN1; AWARD-CHN2	766	381	Glycaemic biomarkers	Greater HbA1c reductions in patients with a higher baseline HbA1c	
35112504	Yabe 2022	Meta-analysis	PIONEER 9; PIONEER 10	652	49	Adiposity biomarkers; Glycaemic biomarkers; Background anti-hyperglycaemic therapy	HbA1c reductions increased as baseline HbA1c increased. There were no other patterns between the variables investigated and HbA1c changes.	
27265893	Wysham 2016	Meta-analysis	AWARD 1 - 6	2806	0	Age; Sex; Diabetes duration; Ethnicity; Adiposity biomarkers;	Higher baseline HbA1c was associated with greater HbA1c reduction. Age ≤65 years, lower FSG	

PMID	Author (year)	Study type	Data Source	Intervention (n)	Comparator (n)	Biomarkers examined	Significant interactions	Notes
						Glycaemic biomarkers; Kidney function biomarkers; History of CVD; Fasting C peptide; Fasting serum insulin	level, FSI level ≤55 pmol/L and eGFR ≤100 mL/min/1.73 m^2 were associated with greater decreases in HbA1c, but the effects were very small.	
33404200	Terauchi 2020	Meta-analysis	LixiLan JP-O1; LixiLan JP-O2; LixiLan JP-L	835	516	Age; Adiposity biomarkers; Glycaemic biomarkers; Background anti-hyperglycaemic therapy	None	
27412701	Shomali 2017	Meta-analysis	LEAD 1 - 6; 1860-LIRA-DPP-4	2698	524	Ethnicity	None	
28573708	Shaw 2017	Meta-analysis	HEELA; EUREXA; NCT00577824; NCT00082381; Nauck 2006; Moretto 2008; NCT00434954; Apovian 2010; DURATION 2; Davies 2012; DURATION-3; DURATION-4; NCT00917267	2355	0	Age; Sex; Diabetes duration; Ethnicity; Adiposity biomarkers; Glycaemic biomarkers; Kidney function biomarkers; Background anti-hyperglycaemic therapy	Baseline HbA1c correlated with change in HbA1c. Asian ethnicity and older age were also significantly associated with high glycaemic response to exenatide twice daily.	
26679282	Seufert 2016	Meta-analysis	LEAD 1-6; Lira-DPP-4i	2689	519	Diabetes duration	None	
29748996	Petri 2018	Meta-analysis	SUSTAIN 1-3; SUSTAIN-Japan OAD combination	1935	1048	Adiposity biomarkers; Glycaemic biomarkers; Sex	Greater effects of semaglutide on HbA1c in the 10% of participants with the lowest body weight than in those with the 10% highest weight.	
26662611	Montanya 2015	Meta-analysis	LEAD 1-6; Lira-DPP-4i	2698	524	Adiposity biomarkers	A modest, clinically non-relevant, association between baseline BMI and HbA1c reduction (10kg/m^2 increase in baseline BMI corresponds with a 1.1mmol/mol greater HbA1c reduction).	
29603872	Mathieu 2018	Meta-analysis	AWARD 1; AWARD 3; AWARD 6	817	0	HOMA2-%B (low, middle and high tertiles)	The low tertile of HOMA2-%B coring individuals experienced larger reductions in HbA1c compared to the high tertile when treated with dulaglutide, however this effect was removed when baseline HbA1c was included as a	

PMID	Author (year)	Study type	Data Source	Intervention (n)	Comparator (n)	Biomarkers examined	Significant interactions	Notes
							covariate. Greater decreases in fasting blood glucose and greater increases in fasting C-peptide were observed in the lowest HOMA2-%B tertile.	
22193143	Henry 2011	Meta-analysis	NA	NA	NA	Glycaemic biomarkers	Reductions in HbA1C levels and HbA1c goal attainment were greater in groups with higher baseline A1C values.	
31055780	Gentilella 2019	Meta-analysis	AWARD 1; AWARD 5; AWARD 6	2040	315	Glycaemic biomarkers	Greater HbA1c reductions in patients with higher baseline HbA1c values.	
28817231	Gallwitz 2017	Meta-analysis	AWARD 1-6; AWARD 8	3375	0	Sex; Diabetes duration; Glycaemic Biomarkers	Greater HbA1c and FBG reductions in patients with a higher baseline HbA1c.	
26594250	Eto 2015	Meta-analysis	GetGoal-Duo1; GetGoal-L; GetGoal-L-Asia	662	0	Adiposity biomarkers	T2D patients in the lowest BMI group relative to those in the highest BMI group had a smaller reduction in HbA1c.	
31769496	DeSouza 2020	Meta-analysis	SUSTAIN 1-5; SUSTAIN 7	3074	0	Ethnicity	None	
26936426	Davidson 2016	Meta-analysis	LEAD 3; LEAD 4; LEAD 6; 1860-LIRA-DPP-4	1755	642	Ethnicity	None	
21700561	Davidson 2011	Meta-analysis	LEAD 1-6	NA	NA	Kidney function biomarkers	None	
28303626	Bonadonna 2017	Meta-analysis	GetGoal-M; GetGoal-P; GetGoal-S	546	0	HOMA-B Index (Low vs High)	None	
27767249	Blonde 2017	Meta-analysis	GetGoal-M; GetGoal-L; GetGoal-Mono; GetGoal-S; GetGoal-P; GetGoal-Duo 1; GetGoal-X; GetGoal-F1; GetGoal-M-Asia; GetGoal-L-Asia	2493	1465	Age; Sex; Diabetes duration; Adiposity biomarkers; Glycaemic biomarkers; Background anti-hyperglycaemic therapy	Higher baseline HbA1 was predictive of increased HbA1c reduction.	
35330424	Kyriakidou 2022	Observational	Medical records	116	0	Age; Sex; Diabetes duration; Adiposity biomarkers; Glycaemic biomarkers; Background anti-hyperglycaemic therapy; Genetics	Higher baseline HbA1c and lower baseline weight were associated with better glycaemic response to liraglutide.	*CTRB1/2* rs7202877 polymorphism not associated with differential glycaemic response.
29076038	Berkovic 2017	Observational	Six tertiary and secondary	207	0	Sex; Glycaemic biomarkers; Cardiovascular	Independent predictors of durability of HbA1c	

PMID	Author (year)	Study type	Data Source	Intervention (n)	Comparator (n)	Biomarkers examined	Significant interactions	Notes
			hospital centres in Croatia			biomarkers; Background anti-hyperglycaemic therapy	reduction were initial BMI, HbA1c, systolic BP, and cholesterol. Female gender and shorter duration of diabetes were independent predictors of greater HbA1c reduction.	
31240562	Yoo 2019	Observational	Asan Medical Center, Republic of Korea	234	0	Age; Sex; Diabetes duration; Adiposity biomarkers; Glycaemic biomarkers; Kidney function biomarkers; Dyslipidaemia; Fasting C peptide	Baseline HbA1c was a significant predictor of glycaemic response to dulaglutide.	
30883264	Yu 2019	Observational	NA	285	0	Genetics	The variant allele T of rs10305420 within the *glpR* gene was associate with a 0.4% smaller HbA1c reduction after 6 month of exenatide treatment	
35383100	Yale 2022	Observational	SURE Canada; SURE Denmark/Sweden; SURE Switzerland; SURE UK	1212	0	Age; Diabetes duration; Adiposity biomarkers; Glycaemic biomarkers; Previous GLP1RA use	Larger reductions of HbA1c in GLP-1RA-naïve versus GLP-1RA switchers; larger reductions in HbA1c for patients with higher versus lower baseline HbA1c.	
32176827	Wang 2020	Observational	Hospital of Xuzhou Medical University, Xuzhou, China	148	0	Age; Sex; Diabetes duration; Adiposity biomarkers; Glycaemic biomarkers; Background anti-hyperglycaemic therapy; Smoking status; Alcohol intake; Family history of diabetes; Fasting serum insulin; Postprandial serum insulin; HOMA-IR; HOMA-B	Baseline HbA1C and duration of diabetes were identified as predictors of HbA1C reduction.	
NA	Thong 2015	Observational	Association of British Clinical Diabetologists Nationwide Liraglutide Audit	937	0	Diabetes duration; Background anti-	Insulin use and longer diabetes duration, but not the number of OADs taken,	

PMID	Author (year)	Study type	Data Source	Intervention (n)	Comparator (n)	Biomarkers examined	Significant interactions	Notes
						hyperglycaemic therapy	predicted a smaller glycaemic response to liraglutide.	
24246138	Thong 2014	Observational	Specialist diabetes centres in the UK	116	0	Age; Sex; Diabetes duration; Ethnicity; Adiposity biomarkers; Glycaemic biomarkers; Kidney function biomarkers; Background anti-hyperglycaemic therapy; Urinary C peptide creatinine ratio	Postprandial urinary C-peptide creatinine ratios before and during liraglutide treatment were weakly associated with the glycaemic response to treatment.	
29527621	Simioni 2018	Observational	ReaL	1325	0	Age; Sex; Diabetes duration; Adiposity biomarkers; Glycaemic biomarkers; Kidney function biomarkers; Background anti-hyperglycaemic therapy; Presence of diabetes complications; Hypertension; Dyslipidaemia	Higher baseline HbA1c and shorter T2D duration were predictive of better glycaemic response to liraglutide.	
28983857	Nunes 2017	Observational	Optum's electronic health records database	5361	0	Ethnicity	None	
27889301	McAdam-Marx 2016	Observational	National electronic medical record data	5141	0	Glycaemic biomarkers; Background anti-hyperglycaemic therapy	Greater HbA1c reductions occurred in insulin-naive patients with baseline HbA1c ≥7.0%.	
31920354	Lee 2019	Observational	NA	120	0	Age; Sex; Diabetes duration; Adiposity biomarkers; Glycaemic biomarkers; Kidney function biomarkers; Cardiovascular biomarkers; History of CVD; Fasting C peptide; ALT; AST	Higher baseline HbA1c was associated with a greater reduction in HbA1c.	
25626486	Lapolla 2015	Observational	Outpatient units in Veneto Region, Italy	481	0	Age; Sex; Diabetes duration; Adiposity biomarkers; Cardiovascular	HbA1c reduction did not differ among baseline BMI classes. HbA1c <7% goal attainment was	

PMID	Author (year)	Study type	Data Source	Intervention (n)	Comparator (n)	Biomarkers examined	Significant interactions	Notes
						biomarkers; Background anti-hyperglycaemic therapy; Glycaemic biomarkers; Previous anti-hyperglycaemic drug use	predicted by previous metformin monotherapy and insulin naivety.	
29341370	Gorgojo-Marti-nez 2017	Observational	Four tertiary Spanish hospitals	148	0	Age; Sex; Diabetes duration; Adiposity biomarkers; Glycaemic biomarkers; Kidney function biomarkers; Cardiovascular biomarkers; History of CVD; Background anti-hyperglycaemic therapy; Microvascular complications; Smoking status; AST; ALT; Background antihypertensive and lipid-lowering drug use.	Higher HbA1c was associated with increased glycaemic response to exenatide.	
29943854	Gomez-Peralta 2018	Observational	Six Spanish centres	799	0	Age; Sex; Diabetes duration; Adiposity biomarkers; Glycaemic biomarkers; Cardiovascular biomarkers; Background anti-hyperglycaemic therapy	Longer treatment with liraglutide was a predictor of improved HbA1c response, whereas higher baseline HbA1c, longer Type 2 diabetes duration and treatment with insulin were predictors of worse HbA1c response.	
26830854	Gimeno-Orna 2016	Observational	NA	117	0	ALT	Elevated baseline transaminase values and decreased transaminase levels during follow-up are associated to a favourable glycaemic response to GLP-1 RAs	
32753164	Dalmazi 2020	Observational	An outpatient diabetes clinic in Italy	186	0	Age; Sex; Diabetes duration; Adiposity biomarkers; Glycaemic biomarkers; Kidney function biomarkers;	Predictors of adequate glycaemic response were shorter diabetes duration and not switching to a different GLP-1RA, respectively	

PMID	Author (year)	Study type	Data Source	Intervention (n)	Comparator (n)	Biomarkers examined	Significant interactions	Notes
						Cardiovascular biomarkers; Background anti-hyperglycaemic therapy		
29386249	Dennis 2018	Observational	PRIBA; CPRD	4803	0	Adiposity biomarkers; Cardiovascular biomarker; Markers of insulin resistance	No evidence of an association between any marker of insulin resistance and 6-month glycaemic response to GLP-1 receptor agonists; no evidence for a difference in response to GLP-1 receptor agonists across the obesity and triglyceride defined subgroups.	
25245811	Chitnis 2014	Observational	General Electric Centricity electronic medical records database	3005	0	Adiposity biomarkers	None	
29241884	Brekke 2017	Observational	Independent administrative claims databases associated with Optimum and HealthCore in the United States	3773	0	Age; Sex; Adiposity biomarkers; Glycaemic biomarkers; Background anti-hyperglycaemic therapy; Cardiovascular Biomarkers; Geographic location; Quan-modified Charlson comorbidity index score; Specialty of prescribing physician; Diabetes-related hospitalisation; Number of ambulance visits; Neuropathy; Retinopathy; Mental illness; Polypharmacy; Hypoglycaemic event; Improving weight control at baseline.	Polypharmacy and hypoglycaemia were associated with greater HbA1c reductions in response to liraglutide. Individuals 18 to 39 years and those with HbA_{1c} of 7.0% to less than 8.0% had higher persistence with liraglutide.	
32574827	Berra 2020	Observational	5 diabetes centres of the Milan (Italy)	626	0	Age; Diabetes duration; Glycaemic biomarkers; Pre-study medication	Predictors of the achievement of HbA1c≤7.0 % were low baseline HbA1c and short duration of diabetes. Neither sex nor age had significant effects	

PMID	Author (year)	Study type	Data Source	Intervention (n)	Comparator (n)	Biomarkers examined	Significant interactions	Notes
							on any clinical or laboratory outcome.	
23630427	Anichini 2013	Observational	5 diabetes outpatient clinics in Tuscany, Italy	315	0	Sex; Diabetes duration; Adiposity biomarkers; Glycaemic biomarkers; Background anti-hyperglycaemic therapy; B-cell function biomarkers	One-year glycaemic target response was associated with high baseline HbA1c levels and longer diabetes duration among males; concomitant metformin therapy was a predictor of better glycaemic targets among females.	
24877253	Carrington 2014	Observational	Medical records in a specialist diabetes clinic	446	0	Diabetes duration; Glycaemic biomarkers; Background anti-hyperglycaemic therapy; GLP1RA treatment length	Higher baseline HbA1c, longer duration of diabetes, longer exenatide use, and use of insulin or sulfonylureas at study end predicted better glycaemic response.	
33801192	Mirabelli 2021	Observational	Unit of Endocrinology and Diabetes at Hospital “Pugliese-Ciaccio” in Catanzaro, Italy	126	0	Glycaemic biomarkers	Higher baseline HbA1c was a predictor of HbA1c reduction ≥ 0.5%.	

Abbreviations: alanine transaminase (ALT), asparttate transaminase (AST), fibroblast growth factor 21 (FGF21), fasting serum glucose (FSG), fasting serum insulin (FSI), Homeostasis Model Assessment 2 - %B (beta cell; HOMA2-%B), Homeostasis Model Assessment - B (beta cell; HOMA-B), Homeostasis Model Assessment - IR (insulin resistance; HOMA-IR), oral antidiabetic drugs (OAD).

Supplementary Table 9: Individual CVOT and renal outcome trials for SGLT2i included in meta-analysis studies

Trial name	Main paper PMID	ClinicalTrials.gov	Population	Interventions	Primary outcome	N	% with T2D	Median fu, years
EMPA-REG	26378978	NCT01131676	Established CVD	Empagliflozin 25mg vs. 10mg vs. placebo	3-point MACE	7,020	100%	3.1
CANVAS*	28605608	NCT01032629	Established CVD or high CVD risk	Canagliflozin 300mg vs. 100mg vs. placebo	3-point MACE	4,330	100%	5.7
CANVAS-R*	28605608	NCT01989754	Established CVD or high CVD risk	Canagliflozin 300mg vs. 100mg vs. placebo	Progression of albuminuria	5,812	100%	2.1
DECLARE	30415602	NCT01730534	Established CVD or high CVD risk	Dapagliflozin 10 mg vs. placebo	3-point MACE	17,160	100%	4.2
CREDENCE	30990260	NCT02065791	eGFR 30-90 and albuminuria	Canagliflozin 100mg vs. placebo	Composite renal	4,401	100%	2.6
VERTIS-CV	32966714	NCT01986881	Established CVD	Ertugliflozin 15mg vs. 5mg vs. placebo	3-point MACE	8,246	100%	3.5
DAPA-HF	31535829	NCT03036124	Established HF	Dapagliflozin 10 mg vs. placebo	HF composite #1	4,744	42%	1.5
SCORED	33200891	NCT03315143	eGFR 25-60	Sotagliflozin 200-400mg vs. placebo	HF composite #1	10,584	100%	1.3
EMPEROR-P	34449189	NCT03057951	Established HF	Empagliflozin 10mg vs. placebo	HF composite #2	5,988	49%	2.2
SOLOIST-WHF	33200892	NCT03521934	Recent HF hospitalization	Sotagliflozin 200-400mg vs. placebo	HF composite #3	1,222	100%	0.8
EMPEROR-R	32865377	NCT03057977	Established HF	Empagliflozin 10mg vs. placebo	HF composite #2	3,730	50%	1.3
DAPA-CKD	32970396	NCT03036150	eGFR 25-75	Dapagliflozin 10 mg vs. placebo	Composite renal	4,304	68%	0.75

3-point MACE: cardiovascular death, non-fatal MI, non-fatal stroke
HF composite #1: hospitalization or an urgent visit with intravenous therapy for heart failure or cardiovascular death
HF composite #2: hospitalization for heart failure or cardiovascular death
HF composite #3: hospitalization or an urgent visit for heart failure or cardiovascular death
*Typically analyzed together

Supplementary Table 10: Individual CVOT trials for GLP1RA included in meta-analysis studies

Trial name	Main paper PMID	ClinicalTrials.gov	Population	Interventions	Primary outcome	N	% with T2D	Median fu, years
ELIXA	26630143	NCT01147250	Established CVD	Lixisenatide 1.8 mg vs. placebo	4-point MACE	6,068	100%	2.1
EXSCEL	28910237	NCT01144338	General T2D	Exenatide 2 mg weekly vs. placebo	3-point MACE	14,752	100%	3.2
HARMONY	30291013	NCT02465515	Established CVD	Albiglutide 30–50 mg weekly vs placebo	3-point MACE	9463	100%	1.6
LEADER	27295427	NCT01179048	Established CVD or high CVD risk	Liraglutide 1.8 mg vs.placebo	3-point MACE	9,340	100%	3.8
PIONEER-6	31185157	NCT02692716	Established CVD or high CVD risk	Semaglutide 14mg daily vs placebo	3-point MACE	3,183	100%	1.3
SUSTAIN 6	28249135	NCT01720446	Established CVD/HF/CKD or high CVD risk	Semaglutide 0.5 mg vs. 1.0 mg vs. placebo	3-point MACE	3,297	100%	2.1
REWIND	31189511	NCT01394952	Established CVD or high CVD risk	Dulaglutide 1.5 mg weekly vs. placebo	3-point MACE	9,901	100%	5.4

3-point MACE: cardiovascular death, non-fatal MI, non-fatal stroke
4-point MACE: as above plus hospitalisation for unstable angina

Supplementary Figure 1: Heat Map of JBI Quality Assessment for included SGLT2i studies

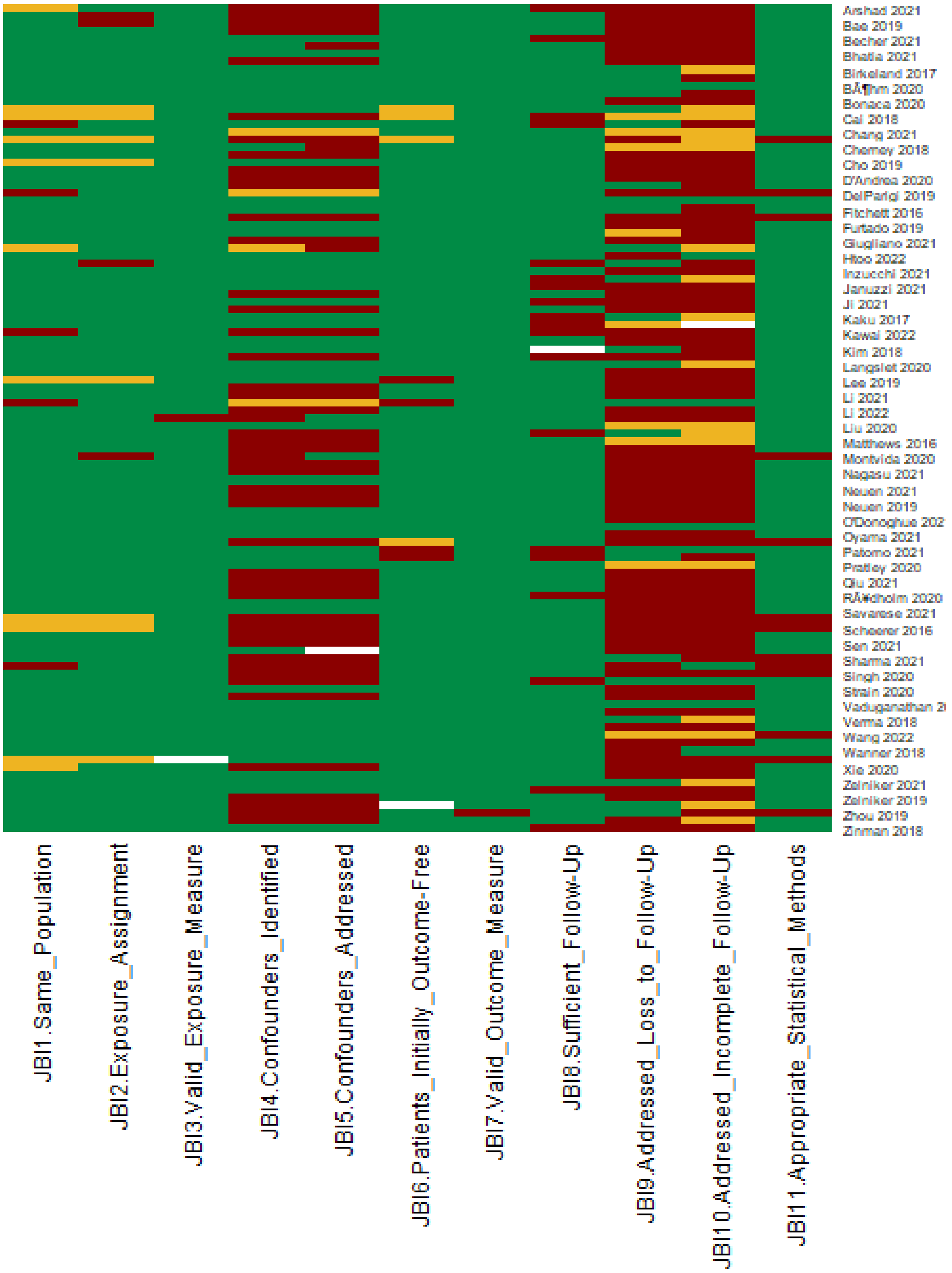

Supplementary Figure 2: Heat Map of JBI Quality Assessment for included GLP1RA studies

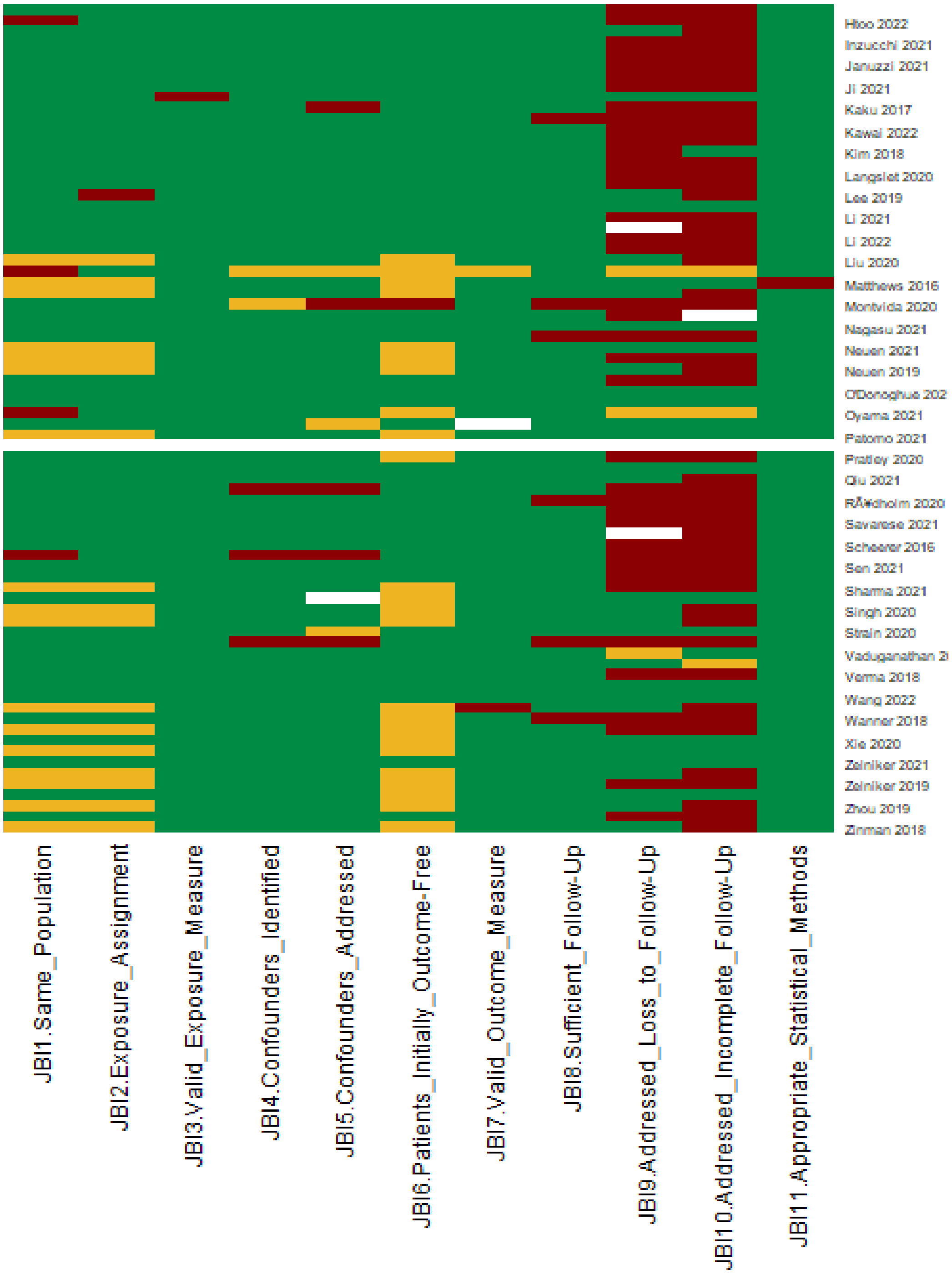

Supplement: Supplementary file 1 — Supplementary Information [file 43856_2023_359_MOESM1_ESM.pdf]
