# Supplementary material for: Treatment effect heterogeneity following type 2 diabetes treatment with GLP1-receptor agonists and SGLT2-inhibitors: a systematic review
Source: Commun Med (Lond). 2023 Oct 5;3:131. doi: 10.1038/s43856-023-00359-w (PMC10551026; doi:10.1038/s43856-023-00359-w)
Supplement: Supplementary file 3 — Reporting Summary [file 43856_2023_359_MOESM3_ESM.pdf]

nature portfolio

Corresponding author(s): Dr John M Dennis

Last updated by author(s): Sep 15, 2023

Reporting Summary

Nature Portfolio wishes to improve the reproducibility of the work that we publish. This form provides structure for consistency and transparency in reporting. For further information on Nature Portfolio policies, see our Editorial Policies and the Editorial Policy Checklist.

Statistics

For all statistical analyses, confirm that the following items are present in the figure legend, table legend, main text, or Methods section.

n/a	Confirmed	
☒	☐	The exact sample size (n) for each experimental group/condition, given as a discrete number and unit of measurement
☒	☐	A statement on whether measurements were taken from distinct samples or whether the same sample was measured repeatedly
☒	☐	The statistical test(s) used AND whether they are one- or two-sided<br>*Only common tests should be described solely by name; describe more complex techniques in the Methods section.*
☒	☐	A description of all covariates tested
☒	☐	A description of any assumptions or corrections, such as tests of normality and adjustment for multiple comparisons
☒	☐	A full description of the statistical parameters including central tendency (e.g. means) or other basic estimates (e.g. regression coefficient) AND variation (e.g. standard deviation) or associated estimates of uncertainty (e.g. confidence intervals)
☒	☐	For null hypothesis testing, the test statistic (e.g. F, t, r) with confidence intervals, effect sizes, degrees of freedom and P value noted<br>*Give P values as exact values whenever suitable.*
☒	☐	For Bayesian analysis, information on the choice of priors and Markov chain Monte Carlo settings
☒	☐	For hierarchical and complex designs, identification of the appropriate level for tests and full reporting of outcomes
☒	☐	Estimates of effect sizes (e.g. Cohen's d, Pearson's r), indicating how they were calculated

Our web collection on statistics for biologists contains articles on many of the points above.

Software and code

Policy information about availability of computer code

Data collection	Covidence (management software for systematic reviews)
Data analysis	Covidence (management software for systematic reviews). No statistical analysis performed.

For manuscripts utilizing custom algorithms or software that are central to the research but not yet described in published literature, software must be made available to editors and reviewers. We strongly encourage code deposition in a community repository (e.g. GitHub). See the Nature Portfolio guidelines for submitting code & software for further information.

Data

Policy information about availability of data

All manuscripts must include a data availability statement. This statement should provide the following information, where applicable:

- Accession codes, unique identifiers, or web links for publicly available datasets
- A description of any restrictions on data availability
- For clinical datasets or third party data, please ensure that the statement adheres to our policy

Template data collection forms and the data extracted from included studies are available upon request. All studies identified by our search protocol are detailed in Supplementary Tables 1-8.

Research involving human participants, their data, or biological material

Policy information about studies with human participants or human data. See also policy information about sex, gender (identity/presentation), and sexual orientation and race, ethnicity and racism.

Reporting on sex and gender	Both males and females were included in the systematic review, based on sex as reported in the included studies. Sex was considered as a treatment effect modifier.
Reporting on race, ethnicity, or other socially relevant groupings	People of all ethnicities were included in the systematic review, based on ethnicity / race as reported in the included studies. Ethnicity was considered as a treatment effect modifier.
Population characteristics	Included studies were selected based on the pre-specified Prospero study protocol (CRD42022303236). Population characteristics of individual studies were not extracted, due to the number of studies (176) included in the review and as we included a mix of study types, including meta-analyses studies.
Recruitment	Included studies were selected based on the pre-specified Prospero study protocol (CRD42022303236), and we considered a wide range of study designs: full-text English-language publications of RCTs, meta-analyses, post-hoc analyses of RCTs, pooled cohort analyses, prospective and retrospective observational analyses. To avoid selection bias we applied broad criteria as detailed in the paper - these are unlikely to have led to any systematic bias in the final list of included study or our findings.
Ethics oversight	None - ethics was not required as this manuscript is a systematic review of published studies.

Note that full information on the approval of the study protocol must also be provided in the manuscript.

Field-specific reporting

Please select the one below that is the best fit for your research. If you are not sure, read the appropriate sections before making your selection.

☒ Life sciences ☐ Behavioural & social sciences ☐ Ecological, evolutionary & environmental sciences

For a reference copy of the document with all sections, see nature.com/documents/nr-reporting-summary-flat.pdf

Life sciences study design

All studies must disclose on these points even when the disclosure is negative.

Sample size	Included studies has a minimum sample size >100 for the active drug of interest
Data exclusions	Exclusions were as follows:<br><4-month potential follow up period (chosen pragmatically as a suitable time length over which changes in glycaemic response could be assessed) after initiation of the drug class of interest.<br>Randomized control trials (RCTs) without a comparison against placebo or an active comparator anti-hyperglycaemic drug. Observational studies without a comparator group.<br>Studies without a pre-specified aim of the study to examine heterogeneity in treatment outcome, such as biomarker-treatment interactions, stratified analyses, or heterogeneity-focused machine learning approaches.<br>Studies that did not report differential effects of the drug class on an outcome of interest with respect to a biomarker.<br>Meta-analyses or pooled cohort analyses in which all individual trial or observational cohorts did not met the inclusion criteria stated above.<br><br>We further excluded studies based on the following criteria: studied type 1 or other forms of non-type 2 diabetes; included children/minors; inpatient studies; conference proceeding abstracts, editorials, opinions papers, book chapters, clinical trial registries, case reports, commentaries, narrative reviews, or non-peer reviewed studies; did not adequately adjust for confounders (individual RCTs and observational studies only, this criteria was not applied for meta-analyses and pooled cohort analyses); did not address the question of treatment response heterogeneity for biomarkers of interest.
Replication	Electronic searches were performed in PubMed and Embase by two independent academic librarians in February 2022. Forwards and backwards citation searching was conducted.<br><br>Titles and abstracts were independently screened by pairs of research team members to identify potentially eligible studies; these were then independently evaluated for inclusion in the full-text review. Any discrepancies were discussed with a third author until reaching consensus. Discrepancies were discussed as part of larger group meetings to ensure consistency in decisions across reviewer pairs.
Randomization	Not applicable
Blinding	Not applicable

Reporting for specific materials, systems and methods

We require information from authors about some types of materials, experimental systems and methods used in many studies. Here, indicate whether each material, system or method listed is relevant to your study. If you are not sure if a list item applies to your research, read the appropriate section before selecting a response.

Materials & experimental systems

n/a	Involved in the study
☒	☐ Antibodies
☒	☐ Eukaryotic cell lines
☒	☐ Palaeontology and archaeology
☒	☐ Animals and other organisms
☒	☐ Clinical data
☒	☐ Dual use research of concern
☒	☐ Plants

Methods

n/a	Involved in the study
☒	☐ ChIP-seq
☒	☐ Flow cytometry
☒	☐ MRI-based neuroimaging
